# EUPopLink Country report – Ireland

**DOI:** 10.12688/openreseurope.23707.1

**Published:** 2026-06-30

**Authors:** Rebecca Mc Donnell, Evangelos Fanoulis

**Affiliations:** 1 School of Political Science and Sociology, University of Galway, Galway, County Galway, Ireland; 2 School of Political Science and Sociology, University of Galway, Galway, County Galway, Ireland

**Keywords:** populism, Ireland, Euroscepticism, discourse

## Abstract

Drawing upon party manifestos during the most recent general elections in Ireland (2024), this country report finds limited signs of either populism or Euroscepticism in the country if we measure both phenomena in terms of electoral success. Whereas certain parties adopt a populist rhetoric, this often focalises on topical policy issues such as the housing crisis or increased migration flows, not achieving broad resonance with the public. Similarly, Eurosceptic voices fall on deaf ears due to the predominance of centrist Fianna Fáil and Fine Gael in the Irish political system, both of which are supportive of European integration. The expression of either populist or Eurosceptic discourse remains in the margins of Irish political life, seen as extreme positions whose electoral impact is mitigated by the country’s PR-STV voting system. The study provides no evidence that a populist discourse that deems EU institutions and policies as the enemy of the people is successful in socially constructing the notion of the people in Ireland.

## 1. Introduction

On the European ^1^ Union’s (EU) political stage, Ireland often portrays itself as the EU’s model pupil. The label can be observed through three occurrences: firstly, when Dublin embraced the Single Market, it did so with greater enthusiasm than most cohesion-fund recipients, when compared to Spain or Greece ( European Commission, 2025). Secondly, modern-Irish economic success is attributed to the EU structural transfers that underwrote the “Celtic Tiger” boom; and thirdly, even after the harsh 2010 bailout, successive governments implemented Brussels-backed reforms without prolonged unrest ( Breen, 2012; Fitzgibbons, 2016: 30; Eichengreen, 2010). Additionally, EU membership is framed not only as an economic lifeline but also as an anchor for the Good Friday Agreement, which became all the more apparent for Ireland in the aftermath of Brexit. This relationship between Ireland and the EU is largely regarded within the political discourse as one ensuring structural cohesion in the country’s governance.

Public attitudes mirror that pragmatic calculus but show clear cycles throughout Ireland’s history post-accession. Eurobarometer surveys reported peak approval during the 1990s ( Breen, 2012: 1). Even when trust in the EU’s institutions touched a historic low of 42% in the aftermath of the Eurozone crisis (Spring Eurobarometer, 2011), overall public support for the EU maintained a level of 63%. As years passed, the pendulum swung back. Brexit re-centred the EU as guarantor of an open border, and the COVID-era recovery funds softened memories of austerity. By early 2025, 61% of Irish respondents said they “tend to trust” the EU ( Eurobarometer, 2025). Overall, approval for EU membership in Ireland remains high.

The importance of the EU among political parties in Ireland comes and goes, as sentiments against the EU appear to flare up at certain moments and then recede. For example, the Irish electorate initially rejected the Lisbon Treaty by referendum in 2008, largely based on the issue of sovereignty, with critics of it reporting that the treaty “centralised” power and eroded democracy ( Fitzgibbons, 2016: 42). Controversially, critics would still cite the second referendum vote as evidence of EU-level “pressure” on Irish voters. However, the ratification of the Lisbon Treaty did not trigger any major realignment in how mainstream parties viewed EU membership, as Fianna Fáil and Fine Gael have continued to remain firmly pro-European. What is more, other parties such as Labour, the Greens and the Social Democrats, frame the EU as a lever for social and environmental standards. Populist or hard Eurosceptic voices are fragmented. On the left, Sinn Féin leads the opposition from 2024 onwards but directs most of its populist displeasure toward the domestic “establishment” parties, framing the EU as Ireland’s potential partner rather than an adversary. On the right, the Irish Freedom Party and the National Alliance demand an “Ire-exit”, the Liberty Republic rails against the ‘Green Deal’ and is largely climate-denialist, with all three being electorally marginal. The 2024 General Election thus stands as a negative case for populism: European integration was contested, on the grounds of neutrality and immigration (among the right parties), but hard Eurosceptic parties gained no seats. In any case, high baseline support for the status quo parties, coupled with PR-STV insulation for centrism, and the recent memory of Brexit, continue to shield Ireland from any durable anti-EU mobilisation, at least for the time being.

## 2. Populist political actors

To proceed with this country report, we analyzed manifesto documents from the 2024 General Election in Ireland, refraining from branding parties as inherently “populist”. As classifying Irish political parties on the traditional left-right spectrum is itself a contested task, instead, we offer a general orientation of their ideological positions for the analysis (Weeks, 2024:209–240; CHES, 2024). ^2^ The empirical investigation measured how often, and in what way, the people-versus-elite binary was invoked. Six political parties stood out for their use of populist rhetoric, even if their H-Scores remain negligible under strict “thin-centred” ideational terms ( Mudde, 2004; Hawkins, 2009). Furthermore, the political parties emerging as populist range from mainstream opposition parties to fringe parliamentary parties, with only a few cases of hard Euroscepticism. Findings are introduced here in a pragmatic sense, with parties using populist discourse rather than being populist per se. Starting from the left and moving toward the right, the parties are:

• **Sinn Féin (left-populist)** The Irish republican party Sinn Féin, whose history extends back as far into Irish history as the 1916 Easter Rising, contests elections on both sides of the Irish border. During the ‘Troubles’, a tumultuous time in Irish history, Sinn Féin positioned itself as Eurosceptic toward the UK’s membership of the EEC, and had been opposed to many European Treaties, namely, Maastricht and the Lisbon Treaty ( Kelly, 2023: 140; Pretorius, 2024). Under the previous party leader, Gerry Adams, the party moved from “war politics” to an electoral contender; and since Mary-Lou MacDonald’s accession to party leader Sinn Féin aligns with pro-European ideals, particularly during and after Brexit ( Kelly, 2023: 140). This position is further illustrated in their 2024 manifesto, which frames the EU as a partner while emphasizing a commitment to stand up for Irish interests within the EU institutions ( Sinn Féin, 2024: 152). Domestically, after the financial crash, Sinn Féin is oppositional to and adversarial in its discourse toward establishment parties. It does so in its construction of the “ordinary people” as neglected in the aftermath of austerity and the current housing crisis, which is evident in the party’s 2024 manifesto. Being the leading opposition party in the Dáil after the 2024 European Elections, its two MEPs caucus with ‘The Left’ in the European Parliament.
• **People Before Profit-Solidarity (Marxist/valence populist)** People Before Profit emerged in 2005 when the Socialist Party and local community-activist campaigners allied together. The party was once conceived as an “electoral project” to widen the revolutionary left and shot to prominence during the post-2008 bank-bailout austerity cycle and the mass water-charges protests ( Molyneux, 2022). Contesting elections on both sides of the Irish border, it is now self-defined as “radical left”. The party blends eco-socialism, anti-war agitation and workers’ control, denouncing elite corporate power and domestic “career politicians” for austerity, climate breakdown and Ireland’s NATO drift. Quantitative coding of its 2024 manifesto yielded a negligible H-Score = 0 (2.65%), yet a discursive read of the document has exposed unmistakable people-versus-elite signifiers in the Housing, Health and Cost of Living policies, evidence of a left-populist register layered onto a Marxist core in how they construct “the people”. As of 2024, in the aftermath of the General Election, PBP lost two Dáil seats with only 3 TDs in the 34th Dáil, with one Belfast MLA, and no elected MEPs. During its 2011–14 stint in Brussels, its lone MEP caucused with The Left/GUE-NGL.
• **Aontú (Socially conservative/soft populist)** Launched in 2019 after Peadar Tóibín quit Sinn Féin over abortion policy, Aontú fuses pro-life social conservatism with left-leaning economic demands such as a Living Wage and curbs on corporate landlords. Domestically, it contrasts “common-policy” and “rural communities” with an urban liberal elite ( Aontú, 2024: 22), registering 1.65% populist sentences in its 2024 party manifesto. Broadly speaking, while not explicitly labelling themselves as Eurosceptic, they do hold Eurosceptic values, in that they oppose EU centralism and the creation of an EU army. The party currently holds only two Dáil seats but no MEPs; its only external ties are loose contacts with the faith-inspired European Christian Political Movement.
• **The Irish Freedom Party (Far-right/Right-populist, Eurosceptic)** Founded in 2018 by Hermann Kelly, the group has campaigned and continues to campaign for a full Irish exit from the EU. In the analysis of the 2024 General Election, they oppose vehemently ‘free movement’, a core tenet of European membership, and view mass immigration as a symptom of Irish membership in the EU. The party has not held, and still does not hold, an elected Dáil member, but does hold one elected local council representative, with no official affiliations with any European party group.
• **The National Alliance (Ethnonationalist/Right-populist)** The alliance of ‘Irish People’ and ‘Ireland First’ has been created since the 2024 General Election, a short-lived electoral brigade designed to avoid “splitting the right vote” ( Ryan, 2024). Its ethno-nationalist rhetoric is front and centre during the election cycle across an array of platforms, and in their manifesto, slogans like “Ireland belongs to the Irish” (McQuinn, 2024) are often paired with anti-immigration and anti-NGO voices. The quantitative coding of their manifesto reveals a modest 2.25% of populist share, while qualitative discourse analysis and the examination of policy areas where populist mentions cluster, show a significant increase of populism when there are references to immigration. During the three election cycles that took place in Ireland in 2024, the alliance won no seats and has since collapsed.
• **Liberty Republic (Far-right/single-issue, right-populist)** Formed in 2010 as Direct Democracy Ireland, Liberty Republic was relaunched in 2024 by Ben Gilroy. The self-proclaimed “political service” espouses anti-immigration sentiments, wanting to “deport illegal immigrants” (Liberty Republic, 2024) and takes an anti-European superstate stance, while not opposing EU membership. From its 2024 election manifesto in its section on the Environment appears to be climate-denialist, professing an inflammatory, conspiratorial populist rhetoric. Its modest overall populist share (2.66%) in the manifesto has concentrated on climate change. In any case, the party has made no electoral gains under Liberty Republic and has no official European affiliations.

## 3. Positions on ‘Europe’ and the Populism-Euroscepticism nexus

Euroscepticism, even in its soft version, has not been electorally successful in Ireland. In an analysis of the 17 political parties that participated in the General Election only the Irish Freedom Party and the National Alliance (NA) outwardly label themselves as ‘Eurosceptic’. They would want to see Ireland leave the EU. Six Irish political parties have deployed a populist rhetoric in the General Election of 2024, under a shared constraint: public approval for EU membership remains among the highest in the Union (61%) ( Eurobarometer, 2025). Following, Euroscepticism within the analysis of the 2024 General Election manifestos remains fragmented and unable to substantially impact the public’s vote. Hard fringe parties like the Irish Freedom Party and National Alliance are joined by Aontú on the topic of the 2024 Migration Pact. But only the Irish Freedom Party and the National Alliance question the EU’s authority outright and flirt with “Ire-exit” language. By contrast, Sinn Féin and People Before Profit reserve their European criticisms for specific policy issues. Sinn Féin casts Brussels as a potential partner, criticising on policy rather than European integration, exhibiting elements of soft Euroscepticism. People Before Profit objects to forms of integration along the lines of NATO alignment and the erosion of neutrality yet stops short of branding the EU as a “corrupt elite.” In short, Irish Euroscepticism seems electorally unsuccessful and narrow. Further, it remains ideologically scattered. Those who view themselves as Eurosceptic are right-wing parties that have their concerns in areas like sovereignty and migration, whereas the left deploys only a selective, policy-level critique.

### 3.1 Left-wing challengers

Sinn Féin’s Euroscepticism has shifted along a continuum: once directed squarely at the EU in the first decade of the 21 ^st^ century due to the Lisbon Treaty, Brexit has transformed their stance on Europe ( Kelly, 2023). Brexit presented the prospect of a hard border on the island of Ireland, forcing Sinn Féin to mellow its Eurosceptic stances. Sinn Féin’s manifesto resulted in a H-Score = 0 (1.09%), which was considered populist according to “thin-centred” ideational markers ( Mudde, 2004; Hawkins, 2009: 1050). Being a soft Eurosceptic party, Sinn Féin would want to work within the European institutions to make policy changes, such as in the Common Agricultural Policy (CAP). From a discursive analysis, populist rhetoric is currently directed toward the domestic “FF/FG establishment”, which has supposedly neglected the “ordinary people” (Sinn Féin manifesto, 2024). The enemy, in other words, appears to be elite mismanagement at home, not the EU as such. Brexit moderated the tone of Sinn Féin when referring to the EU; COVID-19’s Recovery Fund was welcomed as proof that social Europe is possible. The party’s position on the war in Ukraine reflects Ireland’s long-standing foreign policy tradition of neutrality and peacebuilding. Sinn Féin’s manifesto condemns the Russian invasion of Ukraine and expresses solidarity with the people of Ukraine, while articulating a multilateral diplomatic resolution involving the United States, the EU, Russia, and Ukraine (2024: 154).

From the manifesto analysis, People Before Profit (PBP) equally shares a H-Score = 0, but their percentage difference compared to Sinn Féin is higher; PBP’s manifesto document presents an overall percentage of 2.65%. The party wears the label of Eurosceptic somewhat uncomfortably in recent times. As party spokesperson on foreign affairs, Gino Kenny TD insisted that the term “Eurosceptic” has been taken over by the far-right, and would consider the party “euro-critical” ( Matthews, 2023). This stance is consistent with the analysis of the 2024 election. Moreover, the party’s history, much like Sinn Féin, has seen fluctuating expressions of Euroscepticism, as the party was more critical of the EU *post* austerity. PBP generally opposes the neoliberal direction of the Union rather than the idea of cooperation across the member-states. Much like Sinn Féin, PBP’s “criticism” of the EU is policy-specific and during times of grievance. For example, during the ‘Eurozone crisis’, PBP called for the option of an euro referendum and later campaigned for a left-wing Brexit (“Lexit”) in the 2016 UK vote ( Kelly, 2023). And by 2024, they no longer urge an Irish withdrawal. This nuance is further reflected in PBP’s manifesto, as it cites the EU standards as a benchmark in certain policy areas that Ireland needs to meet, such as pensions, climate targets and parental leave, yet takes a critical stance when it sees that military neutrality is potentially at stake. In general terms, “crisis” moments merely redirect the party’s critique: the Eurozone bailout produced certain talk of an euro referendum; COVID-19 shifted discourses to vaccine patents; the election of US President Donald Trump appeared to have minimal impact on the party’s position with Europe; and the Ukraine war sharpened opposition to deepening EU–NATO links. Throughout, PBP seeks a radical overhaul of the EU for now, at least concerning areas of Ireland’s neutrality, but not an exit.

### 3.2 Right-wing challengers

Aontú accepts the economic case for integration but regards Brussels as culturally and socially intrusive. Its 2024 platform backs a living wage and greater state intervention while rejecting the EU Migration Pact and condemning “elite agendas” on social policy. The party speaks of a confederal Europe bound by strict subsidiarity: Ireland should cooperate on trade and environmental goals but reserve ultimate authority over life issues, neutrality and fisheries. In practice, Aontú’s Euroscepticism is soft and highly selective. Populist notes surface chiefly when it contrasts “commonsense families and rural communities” with an urban liberal elite, and even then, the adversary is framed more in domestic than transnational terms. Brexit heightened its call for an all-island economy; subsequent crises left its EU stance largely untouched.

On the right, two parties stand that articulate hard, polity-level Euroscepticism, nonetheless their electoral success is so small that does not change the overal picture of populist Euroscepticism/Eurosceptic populism being almost absent in Irish politics. The Irish Freedom Party places full withdrawal, the so-called “Irexit”, at the centre of its appeal. Its 2024 manifesto vows to reclaim “law-making, currency and borders” from “Brussels and London” (IFP, 2024). Brexit is held up as a successful precedent; COVID-19 health passes are cited as proof of EU authoritarianism. The party’s nationalism is civic, in the sense that any citizen embracing Irish sovereignty belongs to “the Irish People”, and the populist framing is purely vertical: unaccountable Eurocrats versus the demos (IFP, 2024: 1).

While the IFP makes civic appeals, the National Alliance fuses populism with nativism. Immigration quotas, refugee relocation and the Green Deal are all condemned as symptoms of “replacement-level policies forced by Brussels” (National Alliance, 2024). The party demands immediate exit from both the EU and the euro, presenting itself as the sole defender of an ethnically defined Irish nation. For the National Alliance, their Euroscepticism is upfront: Brussels is cast as a force that dilutes Irish culture and sovereignty. Formed after the Brexit vote, the alliance now points to Brexit as proof that an “Ire-exit” can be done and urges a similar referendum at home. Its 2024 manifesto brands EU-backed Covid rules “authoritarian over-reach”, but says little about the Ukraine war or Donald Trump’s presidency; instead, campaign speeches borrow familiar “America-First” and anti-globalist talking points.

Liberty Republic offers a different picture altogether: a soft, single-issue Euroscepticism anchored to climate policy. The party’s brief manifesto rails against “unelected global green elites” who will supposedly “cripple Irish industry” through the EU Green Deal. Beyond that dossier, Liberty Republic is largely silent on European matters. Its stance is therefore maximal on one axis, scrap the Green Deal or leave the EU, and agnostic on most others. Populist language is present but narrowly deployed: “taxpayers and small businesses” against regulators.

Crisis politics has affected the fringe parties – The Irish Freedom Party and the National Alliance - much more than the mainstream parties. These right-wing populist actors encouraged Brexit, used pandemic restrictions as evidence of the creation of an EU-superstate, and now warn that the EU is edging Ireland towards a military alliance with the USA. The prospect of a second Trump Administration revived anti-globalist rhetoric in IFP circles; NA activists echo similar themes, while Liberty Republic aligns its climate scepticism with similar debates in the US.

## 4. Responses to Euroscepticism and consequences

For most of the Irish Republic’s post-membership history, the pro-European political mainstream parties, Fianna Fáil, Fine Gael, Labour, the Greens and more recently the Social Democrats, have treated Europe as a pillar of economic success and guardian of the Good-Friday constitutional settlement. Fitzgibbons (2016: 31) calls this the “difficulty of competing with historic EU-related successes”: the modern-Ireland success story is often attributed as a consequence of EU membership. Further, the cohesion-fund windfalls and the bailout back-stop are publicly framed as nation-saving milestones. Reactions to populist Euroscepticism during the 2024 cycle fit neatly into Meguid’s (2005) menu of strategies: ‘dismissive’ (ignore), ‘accommodative’ (engage in competition, take similar stance) or ‘adversarial’ (engage in competition, take opposite stance).

According to the election result tallies, the Irish Freedom Party, National Alliance and Liberty Republic gathered approximately 1.56% of first-preference votes, holding no national seats, therefore the governing parties saw little incentive to engage and launch a pro-EU counter-narrative. Interestingly, ahead of both the European and General Elections in 2024, the RTÉ’s leaders’ debates required either a sitting TD or an outgoing MEP, a rule that excluded all these three small Eurosceptic actors, depriving them of prime-time television oxygen. Further, the mainstream parties simply repeated the growth-and-peace narrative in campaigns before the European election and the General Election, primarily concerned with domestic policy issues like Housing and the Cost-of-Living crisis, leaving radical criticisms of the Migration Pact or the Green Deal unanswered. This can be viewed as a classic dismissive strategy in accordance to Meguid’s ‘Expanded Toolkit’: “ignore challengers whose salience appears negligible” ( Meguid, 2005: 349). Hard Eurosceptics such as the National Alliance and the Irish Freedom Party got little to no attention from the pro-European mainstream parties. Fianna Fáil and Fine Gael rarely answered their claims or responded to their rhetoric; coverage of these Eurosceptic parties came from the media. Otherwise, mainstream/government parties appeared to have defended neutrality and EU cohesion funds, not in response to such Eurosceptic voices, but because these are the positions they take in general.

The net policy impact of the spiral of populism and Euroscepticism in 2024 is therefore negligible. None of the six parties that used populist language won leverage in government formation; Ireland’s EU vote in the Council of EU or European Parliament remains firmly pro-European. Even Sinn Féin, whose position with Europe has shifted positively in the aftermath of Brexit, deems now Brussels as a partner to be reshaped, not to keep distance from. For the far-right populist parties identified in this report, Euroscepticism appears opportunistic. With no seats, these parties cannot obstruct EU bills or ratifications; their rallies in Ireland generate headlines but cannot produce any legislative amendments. If anything, the result of these political actors has translated into a social-media auditing unit with An Garda Síochana, created after anti-immigrant protests, an indirect, securitising consequence ( Gallagher, 2024).

## Conclusion

At the end of the day, Euroscepticism in Ireland appears to exist without populism and the reverse stands as well. The language of soft Eurosceptic groups is interest-based, not a “moral binary”: they complain about unfair rules rather than paint Brussels as a corrupt elite. This distinction reinforces a core finding of the present study. In Ireland, populist discourse is most visible when socio-economic grievances collide with symbols of national sovereignty, housing versus fiscal rules for Sinn Féin, vaccine patents for PBP, and migration for the right-sovereigntists. Even then, high baseline support for membership and the dampening effect of PR-STV mean that hostile rhetoric rarely matures into a mass Eurosceptic project. Populism remains an idiom of protest, not a roadmap for exit from the EU. As a result, Irish mainstream political actors respond to the few combinedly Eurosceptic and populist voices by ignoring them; only in sectoral corners do they partially accommodate them. For now, the spiral of populism and Euroscepticism in Ireland remains a rhetorical flare, not a driver of institutional change.

## Ethics and consent

Ethical approval and consent were not required for authoring this country report.

## Data Availability

There has been no dedicated dataset compiled for authoring this country report. A summary of our research's results can be accessed on Zenodo Data Repository at: https://doi.org/10.5281/zenodo.20812989

## Footnotes

^1^
The present country report was prepared as part of the EUPopLink COST Action ( Andreadis & Vasilopoulou, 2026) following the EUPopLink Theoretical Framework and guidelines ( Lorimer et al., 2026).

^2^
See “CHES-Europe” (2024) survey data: Fianna Fáil (centre-right), Fine Gael (Christian Democrats), Aontú (conservative), Labour (centre-left), Greens (green), Sinn Féin (left), and Solidarity-People Before Profit (radical-left). See also Weeks (2024) “Parties and the Party System” in *Politics in the Republic of Ireland,* 11 eds. Coakley and Gallagher, who highlight the contested nature of mapping left-right wing politics in Ireland.
