# Supplementary material for: EUPopLink Country report – Ireland
Source: Open Res Eur. 2026 Jun 30;6:222. [Version 1] doi: 10.12688/openreseurope.23707.1 (PMC13560861; doi:10.12688/openreseurope.23707.1)
Supplement: Peer review history

# Reviewer report: Reviewer response for version 1

**DOI:** 10.21956/openreseurope.25665.r77513

**Published:** 2026-08-03

**Authors:** Tim White

**Affiliations:** 1 Xavier University, Cincinnati, Ohio, USA

This article clearly and effectively summarizes the relatively low rates of anti-European Union sentiment in Ireland. It also discusses how the majority of the public in Ireland is also not supportive of populist left wing or right wing parties. The historic support of center-right and center-right parties minimizes the appeal of more extremist parties. Ireland's history as a state that experienced mass migration since the famine in the 19th century also makes it less likely to appeal to anti-immigration sentiment.

 Ireland's relatively low unemployment rate also makes it less likely that voters and the public blame immigrants for loss of jobs in the Irish context. Most realize that Ireland's place in the EU has benefitted Ireland earlier in terms of an agricultural market and more recently in terms of foreign investment, especially in the pharmaceutical, IT, and financial sectors. The complicating factor in Irish support for the EU is its historic support for neutrality which undermines EU security and defense cooperation. Overall, this piece does an excellent job of summarizing Irish attitudes toward the EU and populism.

Is the case presented with sufficient detail to be useful for teaching or other practitioners?

Yes

Is the work clearly and accurately presented and does it cite the current literature?

Yes

If applicable, is the statistical analysis and its interpretation appropriate?

Yes

Are all the source data underlying the results available to ensure full reproducibility?

Yes

Are the conclusions drawn adequately supported by the results?

Yes

Is the background of the case’s history and progression described in sufficient detail?

Yes

Reviewer Expertise:

Irish Politics, culture, and the peace process in Northern Ireland

I confirm that I have read this submission and believe that I have an appropriate level of expertise to confirm that it is of an acceptable scientific standard.

# Reviewer report: Reviewer response for version 1

**DOI:** 10.21956/openreseurope.25665.r76812

**Published:** 2026-07-13

**Authors:** Conor J. Kelly

**Affiliations:** 1 Balliol College,University of Oxford, Oxford, England, UK

This is a helpful and interesting piece. The comments below are on minor points of detail, mostly some suggestions for adding clarity.

 Start of intro: As you show later, as well as portraying itself as a model pupil, Ireland has also been 'described' as a model pupil.

 'What is more, other parties such as Labour, the Greens and the Social Democrats, frame the EU as a lever for social and environmental standards.'...Later you say 'for most of the Irish Republic’s post-membership history, the pro-European political mainstream parties, Fianna Fáil, Fine Gael, Labour, the Greens and more recently the Social Democrats, have treated Europe as a pillar of economic success and guardian of the Good-Friday constitutional settlement.'....just a note that the Green Party opposed Nice and were divided during Lisbon 1. Obviously, the Social Democrats were not around for those referendums. It might be more accurate to say FF/FG have always been very pro-EU, and Ireland's center left parties have been largely supportive too, especially recently. 

 It is of course contestable that the Sinn Féin party of today has a history that 'extends back as far into Irish history as the 1916 Easter Rising'. The modern party re-emerges after the republican split in 1969/1970. In any case, leaving that point aside, Sinn Féin were marginal players in the Rising in 1916, and only took center stage in the years following those events (1918 General Election). I would amend this sentence with something that is less contestable or simply remove it entirely. It adds little to the analysis. You don't mention that FF, FG, or Labour also have histories stretching back to the revolutionary period.

 The final sentence on Sinn Féin could be read as connecting two seemingly unrelated points: 'Being the leading opposition party in the Dáil after the 2024 European Elections, its two MEPs caucus with ‘The Left’ in the European Parliament.'...Sinn Féin first had an MEP sit with this group in 2004 (20 years before the 2024 GE). For clarity I would break up this sentence and make the points separately. 

 'Ireland’s NATO drift' needs a citation, or alternatively, make it clear this is their perspective on the issue not an accepted one across Irish politics or academia.

 PBP having 'one Belfast MLA' could be clearer. A lay reader might assume Belfast was in the Republic. I would say 'one MLA in Northern Ireland' for clarity.

 PBP 'During its 2011–14 stint in Brussels' is confusing...the mandate was 2009-2014. Joe Higgins replaced by Paul Murphy and both sat with GUE-NGL/The Left.

 The National Alliance 'while qualitative discourse analysis and the examination of policy areas where populist mentions cluster, show a significant increase of populism when there are references to immigration'...this is interesting, provide empirical detail if you can.

 'For example, during the ‘Eurozone crisis’, PBP called for the option of an euro referendum and later campaigned for a left-wing Brexit (“Lexit”) in the 2016 UK vote (Kelly, 2023).'...I think you need another source for this. The Kelly 2023 article does not cover PBP.

 Aontú: 'Its 2024 platform backs a living wage and greater state intervention while rejecting the EU Migration Pact and condemning “elite agendas” on social policy'..here and elsewhere when you are citing a specific claim from a manifesto, you could include page numbers in brackets so readers can find the relevant part of the manifesto.

 'At the end of the day, Euroscepticism in Ireland appears to exist without populism and the reverse stands as well.'....I would rephrase this for clarity and lose the colloquialism which may confuse non-native English speakers.

 You refer in passing to the role played by PR-STV a few times but I would flesh this out somewhere in the article. It is an interesting point.

Is the case presented with sufficient detail to be useful for teaching or other practitioners?

Yes

Is the work clearly and accurately presented and does it cite the current literature?

Yes

If applicable, is the statistical analysis and its interpretation appropriate?

Yes

Are all the source data underlying the results available to ensure full reproducibility?

Yes

Are the conclusions drawn adequately supported by the results?

Yes

Is the background of the case’s history and progression described in sufficient detail?

Yes

Reviewer Expertise:

Party politics and EU politics.

I confirm that I have read this submission and believe that I have an appropriate level of expertise to confirm that it is of an acceptable scientific standard.
